# Molecular confirmation of *Anopheles gambiae* sensu stricto (Diptera: Culicidae) at multiple locations in southern Ethiopia

**DOI:** 10.1093/jme/tjag173

**Published:** 2026-11-12

**Authors:** Daibin Zhong, Guofa Zhou, Teshome Degefa, Werissaw Haileselassie, Ashenafi Abossie, Ming-Chieh Lee, Dawit Hawaria, Xiaoming Wang, Fekadu Massebo, Delenasaw Yewhalaw, Guiyun Yan

**Affiliations:** Joe C. Wen School of Population & Public Health, University of California at Irvine, Irvine, CA, USA; Joe C. Wen School of Population & Public Health, University of California at Irvine, Irvine, CA, USA; Faculty of Health Sciences, Jimma University, Jimma, Ethiopia; College of Health Sciences, Addis Ababa University, Addis Ababa, Ethiopia; College of Medicine and Health Sciences, Arba Minch University, Arba Minch, Ethiopia; Joe C. Wen School of Population & Public Health, University of California at Irvine, Irvine, CA, USA; School of Public Health, Hawassa University, Hawassa, Ethiopia; Joe C. Wen School of Population & Public Health, University of California at Irvine, Irvine, CA, USA; Department of Biology, Arba Minch University, Arba Minch, Ethiopia; Faculty of Health Sciences, Jimma University, Jimma, Ethiopia; Tropical and Infectious Diseases Research Center (TIRC), Jimma University, Jimma, Ethiopia; Joe C. Wen School of Population & Public Health, University of California at Irvine, Irvine, CA, USA

**Keywords:** malaria vector surveillance, molecular species identification, vector ecology, *Anopheles gambiae* complex, Ethiopia

## Abstract

*Anopheles gambiae* sensu stricto Giles is a major malaria vector in sub-Saharan Africa but has been infrequently documented in Ethiopia, where *Anopheles arabiensis* has historically been considered the predominant member of the *Anopheles gambiae* complex. We investigated *Anopheles* species composition during larval surveillance at 8 sites in southern and southwestern Ethiopia during 2025–2026. Larvae species were identified using multiplex PCR and species-specific TaqMan quantitative PCR (qPCR), with selected specimens further characterized by internal transcribed spacer 2 (ITS2) and mitochondrial cytochrome *c* oxidase subunit 1 (*cox1*) sequencing. Among 503 larvae from 94 molecularly sampled aquatic habitats, 216 were identified as *An. arabiensis*, 158 as *An. stephensi*, and 40 as *An. gambiae* s.s.; 89 remained unresolved. *Anopheles gambiae* s.s. was detected at 4 geographically distinct sites: Soddo, Birbir, Dila, and Arba Minch. All 40 specimens were concordantly identified by multiplex PCR and qPCR, and their identical ITS2 sequences matched *An. gambiae* s.s. reference sequences from Kenya and Tanzania. *Anopheles gambiae* s.s. was detected in 18 molecularly sampled habitats (12 rural and 6 peri-urban), whereas *An. stephensi* was detected in 35 habitats (27 urban, 6 peri-urban, and 2 rural). Because sampling was not designed to estimate relative abundance or habitat preference, these ecological patterns are descriptive. These findings confirm *An. gambiae* s.s. at multiple locations in southern Ethiopia and support the use of molecular identification for improved characterization of malaria vector species composition.

## Introduction

Malaria remains a major public health challenge in Ethiopia, where transmission is geographically heterogeneous and influenced by rainfall, elevation, land use, and ecological variation. Following earlier progress in malaria control, Ethiopia experienced a substantial resurgence, with more than 7.3 million confirmed malaria cases and 1,157 deaths reported in 2024 (Sasie et al. 2026). Recent investigations have highlighted persistent regional differences in malaria burden, emerging insecticide resistance, and operational challenges affecting malaria surveillance and control (Woldesenbet et al. 2025, Sasie et al. 2026, Teshome et al. 2026). Accurate identification of malaria vector species and characterization of their geographic occurrence and ecology are therefore important for informing vector surveillance and locally appropriate control strategies.

*Anopheles arabiensis* Patton has historically been regarded as the principal malaria vector in Ethiopia and the predominant member of the *Anopheles gambiae* species complex (Sinka et al. 2012, Coetzee et al. 2013, PMI 2024). In contrast, *Anopheles gambiae* Giles sensu stricto (*An. gambiae* s.s.), a major malaria vector in sub-Saharan Africa (Gillies and Coetzee 1987, Takken et al. 2024), has been reported less frequently in Ethiopia. Earlier molecular evidence documented its occurrence in south-central Ethiopia (Kinfe and Tekie 2019), and a recent whole-genome sequencing study confirmed its presence in Gambella and Benishangul-Gumuz in western Ethiopia (Gemechu et al. 2026). These findings establish that *An. gambiae* s.s. occurs in Ethiopia, although its geographic distribution and local occurrence remain incompletely characterized. Molecular surveillance across additional locations is needed to improve understanding of the composition and distribution of the *An. gambiae* complex.

Accurate identification of members of the *An. gambiae* complex is challenging because several sibling species cannot be reliably distinguished using conventional morphological characteristics. In addition to* An. arabiensis* and *An. gambiae* s.s., *Anopheles amharicus* Hunt, Wilkerson, and Coetzee has been documented in Ethiopia (Hunt et al. 1998, Coetzee et al. 2013, Demissew et al. 2020, Demissew et al. 2022). Species-specific multiplex PCR and TaqMan quantitative PCR (qPCR) provide complementary approaches for identifying members of the complex (Scott et al. 1993, Walker et al. 2007, Singh et al. 2023), while sequencing of the nuclear ribosomal internal transcribed spacer 2 (ITS2) region offers additional evidence for species confirmation. Mitochondrial cytochrome c oxidase subunit 1 (*cox1*) sequencing can further characterize mitochondrial genetic variation. Combining these approaches can strengthen the interpretation of species occurrence, particularly when investigating species that have been infrequently documented in a geographic region.

The invasive malaria vector *Anopheles stephensi* Liston presents an additional challenge to malaria surveillance and control in Ethiopia. Its establishment in urban and peri-urban environments has increased attention to the diversity of aquatic habitats supporting malaria vectors. Although *An. stephensi* is frequently associated with artificial water-storage containers and other human-modified habitats, its larval habitat distribution may vary across local environments. Characterizing the habitats in which *An. gambiae* s.s. and *An. stephensi* are detected within the same surveillance areas may provide useful baseline information on local vector species composition and identify priorities for subsequent ecological investigations.

In this study, we investigated the occurrence of *An. gambiae* s.s. among mosquito larvae collected from 8 sites in southern and southwestern Ethiopia during 2025–2026 as part of malaria vector surveillance activities under the Ethiopia International Centers of Excellence for Malaria Research (ICEMR) project. We used species-specific multiplex PCR, TaqMan qPCR, and ITS2 sequencing to establish species identity, with *cox1* sequencing providing complementary information on mitochondrial genetic variation. We also characterized the observed larval habitat distributions of *An. gambiae* s.s. and *An. stephensi* using habitat-level collection records. By documenting molecularly confirmed *An. gambiae* s.s. at multiple locations in southern Ethiopia, this study provides additional evidence of its geographic occurrence and contributes to a more complete characterization of malaria vector species composition in Ethiopia.

## Materials and Methods

### Study Sites and Mosquito Collections

Prospective mosquito larvae surveys were conducted at 8 sites in southern and southwestern Ethiopia during 2025–2026 as part of malaria vector surveillance activities under the Ethiopia ICEMR project (Fig. 1). To represent diverse rural, peri-urban, and urban ecological settings, sampling was integrated into the established ICEMR network across 8 specific locations: Soddo, Birbir, Arba Minch, Shashemene, Hawassa, Dila, Bule Hora, and Jimma. These locations were selected within the established ICEMR surveillance network, where prospective larval surveillance was being conducted.

**Fig. 1.**
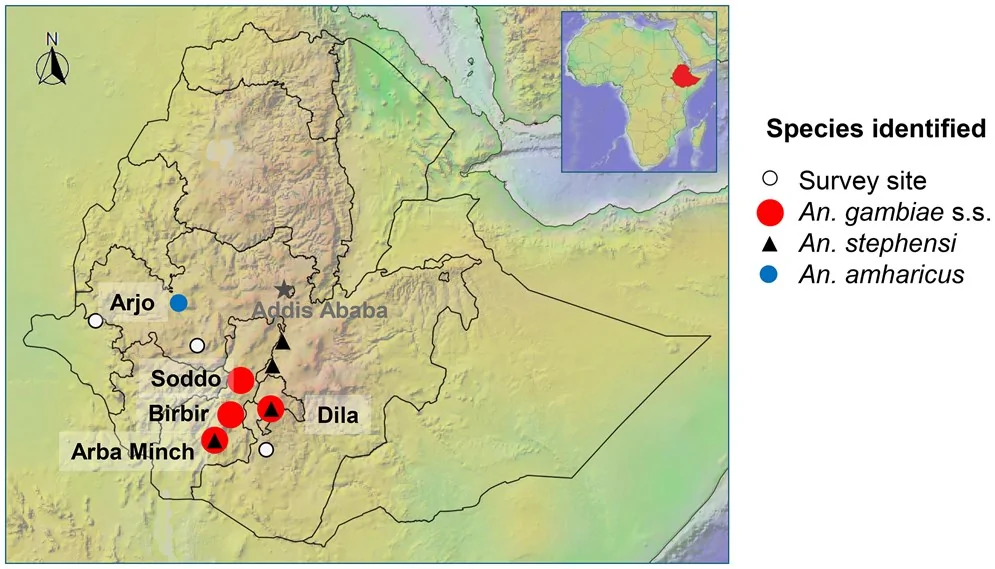
Distribution of mosquito survey sites and detection of *An. gambiae* s.s., *An. amharicus*, and *An. stephensi* in southern and southwestern Ethiopia. Administrative boundaries were obtained from GADM v4.1 (GADM 2022), and the terrain basemap was generated from Natural Earth shaded relief data (Patterson and Kelso 2024).

At each site, all accessible potential mosquito breeding habitats were examined using standard larval dipping methods (WHO 2013). Each habitat was dipped 1 to 20 times using a standard mosquito dipper of 300 ml (Model 320, John W. Hock Company, Gainesville, FL, USA). Natural and human-modified aquatic habitats, including puddles, drainage channels, irrigation-associated pools, ponds, water-storage containers, concrete reservoirs, and other temporary or permanent water bodies were surveyed. Geographic coordinates and elevation were recorded for each sampling location using Open Data Kit (ODK) on tablet devices.

Larvae were morphologically identified to the genus *Anopheles* using standard taxonomic keys (Gillies and De Meillon 1968, Gillies and Coetzee 1987) and individually preserved in 100% ethanol for molecular analysis (Minakawa et al. 1999). Specimens were maintained at ∼4 °C in a cooler during field collection and transport and subsequently stored at −20 °C until molecular analysis.

A total of 236 aquatic habitats were surveyed. Of the 236 aquatic habitats surveyed, 114 contained *Anopheles* larvae, and 94 of these positive habitats were represented by at least 1 larva in the molecular dataset. From habitats containing *Anopheles* larvae, larvae at the second instar or later were selected for molecular analysis, with sampling designed to maximize representation across positive habitats rather than to provide standardized estimates of larval abundance. The final molecular dataset comprised 503 larvae representing 94 aquatic habitats across the 8 prospective survey sites. Each larva was linked to its original habitat identification code for habitat-level analyses.

In addition, 337 archived adult *Anopheles* mosquitoes collected by the Centers for Disease Control and Prevention (CDC) light-trap at Arjo and Gambella during 2017 and 2019 were included as reference specimens for molecular and phylogenetic comparisons. These archived specimens were analyzed separately from the prospective larval collections and were excluded from larval habitat analyses.

### DNA Extraction

Genomic DNA was extracted individually from whole mosquito larvae or from legs dissected from archived adult mosquitoes using a modified enzymatic lysis method (Santos et al. 2018, Makunin et al. 2022). Briefly, ethanol-preserved specimens were rinsed twice in fresh 100% ethanol to remove residual storage ethanol and potential surface contaminants, air-dried, and transferred to 0.5 ml microcentrifuge tubes containing 50 µl extraction buffer consisting of 200 mM Tris-HCl (pH 8.0), 25 mM EDTA (pH 8.0), 0.05% Tween 20, and 0.4 mg/ml Proteinase K. Samples were briefly centrifuged and incubated at 56 °C for 1 h to facilitate tissue digestion and DNA release. Following incubation, samples were centrifuged again, and the supernatant containing genomic DNA was transferred to a 96-well storage plate. DNA lysates were diluted 1:50 by mixing 2 µl of crude extract with 98 µl of molecular-grade water, and 2 µl of diluted DNA was used as template for subsequent PCR and qPCR assays.

### Multiplex PCR Species Identification

Species identification was performed using a modified multiplex PCR assay incorporating previously published primers (Scott et al. 1993, Zhong et al. 2020, Haileselassie et al. 2021, Ali et al. 2022). The assay contained 6 primers (each supplied at 10 µM). The universal forward primer (UN), together with three species-specific reverse primers targeting *An. arabiensis* (AR), *An. gambiae* s.s. (GA), and *An. quadriannulatus* (QDB) was used to differentiate members of the *An. gambiae* complex, whereas the internal transcribed spacer 2 (ITS2A) forward primer and 28S ribosomal DNA reverse primer (28SC) were included for detection of *An. stephensi*. Primer sequences are provided in Supplementary Table S1.

PCR amplification was performed in 17 µl reactions containing 8.5 µl DreamTaq Green PCR Master Mix (2×) (Thermo Fisher Scientific, Waltham, MA, USA), 0.5 µl of each of the 6 primers (10 µM), 2 µl DNA template, and 3.5 µl nuclease-free water. Thermal cycling consisted of an initial denaturation at 95 °C for 3 min, followed by 35 cycles of 94 °C for 30 s, 55 °C for 30 s, and 72 °C for 60 s, with a final extension at 72 °C for 6 min. Amplified products were separated on 2% agarose gels and visualized under UV illumination. Expected diagnostic amplicon sizes were 390 bp for *An. gambiae* s.s., 315 bp for *An. arabiensis*, ∼780/900 bp for *An. amharicus*, and 472 bp for *An. stephensi*. The *An. amharicus* amplification pattern observed in the multiplex assay was confirmed using the UN/QDB primer pair alone.

Positive species controls and a no-template negative control were included in every PCR run. Samples yielding an inconclusive amplification pattern were repeated once using the same DNA extract. Samples that remained inconclusive after repeat testing were recorded as unresolved by multiplex PCR. All 503 prospective larvae and 337 archived adults were examined by multiplex PCR.

### TaqMan qPCR Species Identification

All 503 prospectively collected larvae were additionally analyzed using a multiplex TaqMan qPCR assay for *An. gambiae s.s., An. arabiensis*, and *An. stephensi* (Walker et al. 2007, Singh et al. 2023). The assay utilized primers 623F and 788R to amplify the 28S ribosomal RNA gene region for members of the *An. gambiae* complex, while primers stq_F and stq_R amplified the ribosomal ITS2 region for *An. stephensi*. Species-specific detection was achieved using AG_NED (*An. gambiae* s.s.), AR_FAM (*An. arabiensis*), and ST_VIC (*An. stephensi*) probes. All primer and probe sequences are listed in Supplementary Table S1. The assay did not contain an *An. amharicus*-specific probe.

Reactions were performed in 12 µl volumes containing 6.0 µl TaqMan Fast Advanced Master Mix (2×), 0.5 µl of each probe (2.0 µM working concentration; 83.3 nM final concentration), 0.4 µl of each primer (10 µM working concentration; 333.3 nM final concentration), 2 µl DNA template, and 0.9 µl nuclease-free water. Amplification was performed on a QuantStudio 3 Real-Time PCR Instrument (Applied Biosystems) using the following cycling conditions: 50 °C for 2 min and 95 °C for 2 min, followed by 45 cycles of 95 °C for 3 s and 60 °C for 30 s. Fluorescence signals were analyzed using QuantStudio Design and Analysis software. Positive controls representing *An. gambiae s.s., An. arabiensis*, and *An. stephensi*, together with no-template controls, were included in each run. A sample was considered positive when the corresponding species-specific amplification curve had a cycle threshold (Ct) ≤ 30. Inconclusive reactions were repeated once using the same DNA extract.

Archived adults were analyzed by multiplex PCR but were not routinely analyzed by qPCR; selected archived specimens were subsequently characterized by sequencing.

### ITS2 and *cox1* Amplification and Sanger Sequencing

A total of 97 specimens representing *An. gambiae* s.s. (*n* = 40), *An. arabiensis* (*n* = 26), and *An. amharicus* (*n* = 31) were selected for sequence-based analysis. All 40 *An. gambiae* s.s. specimens detected at the 4 positive prospective survey sites were included. These specimens were subjected to ITS2 sequencing for additional molecular confirmation of species identity and to mitochondrial cox1 sequencing for complementary assessment of genetic relationships.

The nuclear ribosomal ITS2 region was amplified using primers ITS2A and ITS2B (Beebe and Saul 1995), and the mitochondrial *cox1* region was amplified using primers LCO1490 and HCO2198 (Folmer et al. 1994). PCR amplifications were performed in 17 µl volumes containing 8.5 µl DreamTaq™ Green PCR Master Mix (2 ×) (Thermo Fisher Scientific, Waltham, MA, USA), 0.5 µl of each primer (10 µM), 2 µl DNA template, and 5.5 µl nuclease-free water. Thermal cycling consisted of an initial denaturation at 95 °C for 3 min, followed by 35 cycles of 94 °C for 30 s, 55 °C for 30 s, and 72 °C for 60 s, with a final extension at 72 °C for 6 min. Five microliters of each amplified product were examined by electrophoresis on a 2.0% agarose gel.

PCR products showing a single clear band were purified enzymatically using Exonuclease I and shrimp alkaline phosphatase. For each reaction, 8.0 µl PCR product was mixed with 0.3 µl Exonuclease I (20 U/µl) and 1.7 µl shrimp alkaline phosphatase (1 U/µl). The mixture was incubated at 37 °C for 30 min, followed by 80 °C for 15 min to inactivate the enzymes, and then held at 4 °C until sequencing. Purified PCR products were prepared separately for forward and reverse sequencing by combining 3 µl of cleaned PCR product, 3 µl of the corresponding sequencing primer (10 µM), and 12 µl ddH_2_O. Samples were submitted to GENEWIZ (South Plainfield, NJ, USA) for bidirectional Sanger sequencing using the same forward and reverse primers employed for PCR amplification.

Forward and reverse chromatograms were inspected using CodonCode Aligner version 12.0.5. Low-quality terminal bases were trimmed, and overlapping forward and reverse reads were assembled into consensus sequences. All 97 specimens produced ITS2 consensus sequences suitable for analysis. For *cox1*, 84 sequences met the quality-control criteria and were retained, comprising 27 *An. gambiae* s.s., 26 *An. arabiensis*, and 31 *An. amharicus*. Thirteen *An. gambiae* s.s. *cox1* sequences were excluded after sequence quality control.

Identical ITS2 sequences within each species group were represented by a single deposited representative sequence. Sequence lengths, specimen information, sequence/haplotype assignments, and GenBank accession numbers are provided in Supplementary Materials.

### Phylogenetic, Genetic Distance, and Haplotype Analyses

ITS2 consensus sequences were aligned with representative *An. gambiae* complex sequences retrieved from GenBank using BioEdit (Hall 1999). Maximum-likelihood phylogenetic analysis was performed in MEGA12 (Kumar et al. 2024). The Jukes-Cantor nucleotide substitution model was selected based on the lowest Bayesian information criterion (BIC) and corrected Akaike information criterion (AICc) values. Branch support was assessed using 1,000 bootstrap replicates.

For mitochondrial *cox1*, unique haplotypes were identified from the 84 quality-controlled sequences using DnaSP version 6.12.03 (Rozas et al. 2017). Representative haplotypes were analyzed together with reference sequences retrieved from GenBank. ModelTest-NG was used to evaluate nucleotide substitution models, and HKY+I was selected according to both BIC and AICc. Maximum-likelihood phylogenetic reconstruction was performed using PhyML under the HKY+I model, with empirical nucleotide frequencies and the proportion of invariant sites estimated from the data. Branch support was assessed using 1,000 nonparametric bootstrap replicates. *Anopheles quadriannulatus* Theobald (PP747845) was used as the outgroup. A Templeton-Crandall-Sing (TCS) haplotype network based on *cox1* sequences was constructed using PopART (Leigh et al. 2015) to visualize mitochondrial haplotype relationships among *An. gambiae* s.s., *An. arabiensis*, and *An. amharicus*.

Pairwise Kimura 2-parameter (K2P) genetic distances were calculated separately for ITS2 and *cox1*. For ITS2, representative sequences included in the phylogenetic analysis were compared; alignment gaps were excluded from nucleotide-distance calculations and indel differences were considered separately. For *cox1*, distances were calculated among the unique haplotypes included in the phylogenetic analysis.

### Statistical and Habitat-Level Analyses

Molecular identification outcomes were summarized by collection site, species, and aquatic habitat. Because larvae were subsampled from positive habitats to maximize habitat representation and sampling effort was not standardized among habitats or sites, individual larvae were not treated as independent replicates for inference about habitat preference or relative abundance. For ecological summaries, the aquatic habitat was therefore treated as the observational unit. Species detection was defined as the presence of at least 1 molecularly identified specimen of a given species within a habitat. Habitat-level detection frequencies were summarized descriptively by habitat type and environmental setting (rural, peri-urban, and urban). Habitats containing *Anopheles* larvae but not represented in the molecular subsample were not classified as molecularly negative for individual species. Archived adult specimens from Arjo and Gambella were excluded from habitat-level analyses.

## Results

### Molecular Identification of Prospective Larval Collections

A total of 503 *Anopheles* larvae from 94 aquatic habitats at 8 prospective survey sites were analyzed molecularly (Table 1; Fig. 1). These specimens represented a subset of larvae collected from 236 surveyed aquatic habitats, of which 114 contained *Anopheles* larvae (Supplementary Table S2). Molecular identification assigned 216 larvae to *An. arabiensis*, 158 to *An. stephensi*, and 40 to *An. gambiae* s.s.; 89 specimens remained unresolved (Table 1).

**Table 1.** Prospective larval surveillance and molecular identification of *Anopheles* mosquitoes at 8 survey sites in southern and southwestern Ethiopia, 2025–2026

Site	Aquatic habitats surveyed	*Anopheles* positive habitats	Habitats represented in molecular analysis	Larvae molecularly tested	*An. gambiae* s.s.	*An. arabiensis*	*An. stephensi*	Unresolved
**Soddo**	48	24	15	58	31	6	0	21
**Birbir**	16	7	6	41	4	36	0	1
**Arba Minch**	35	26	16	70	3	26	38	3
**Dila**	24	8	8	35	2	2	13	18
**Hawassa**	70	27	27	170	0	69	101	0
**Shashemene**	20	6	6	26	0	20	6	0
**Bule Hora**	11	4	4	20	0	19	0	1
**Jimma**	12	12	12	83	0	38	0	45
**Total**	236	114	94	503	40	216	158	89

Multiplex PCR produced species-specific diagnostic amplicons consistent with *An. gambiae s.s., An. arabiensis*, and *An. stephensi* (Fig. 2). Among the 503 prospectively collected larvae, multiplex PCR and species-specific qPCR produced concordant positive species assignments for 409 specimens, including 40 *An. gambiae* s.s., 214 *An. arabiensis*, and 155 *An. stephensi* (Supplementary Table S3). Five specimens that were inconclusive by multiplex PCR were resolved by qPCR as *An. arabiensis* (*n* = 2) or *An. stephensi* (*n* = 3). The remaining 89 specimens were inconclusive in both assays and were retained as unresolved. Thus, final molecular assignments among the 503 larvae were 40 *An. gambiae* s.s., 216 *An. arabiensis*, 158 *An. stephensi*, and 89 unresolved specimens.

**Fig. 2.**
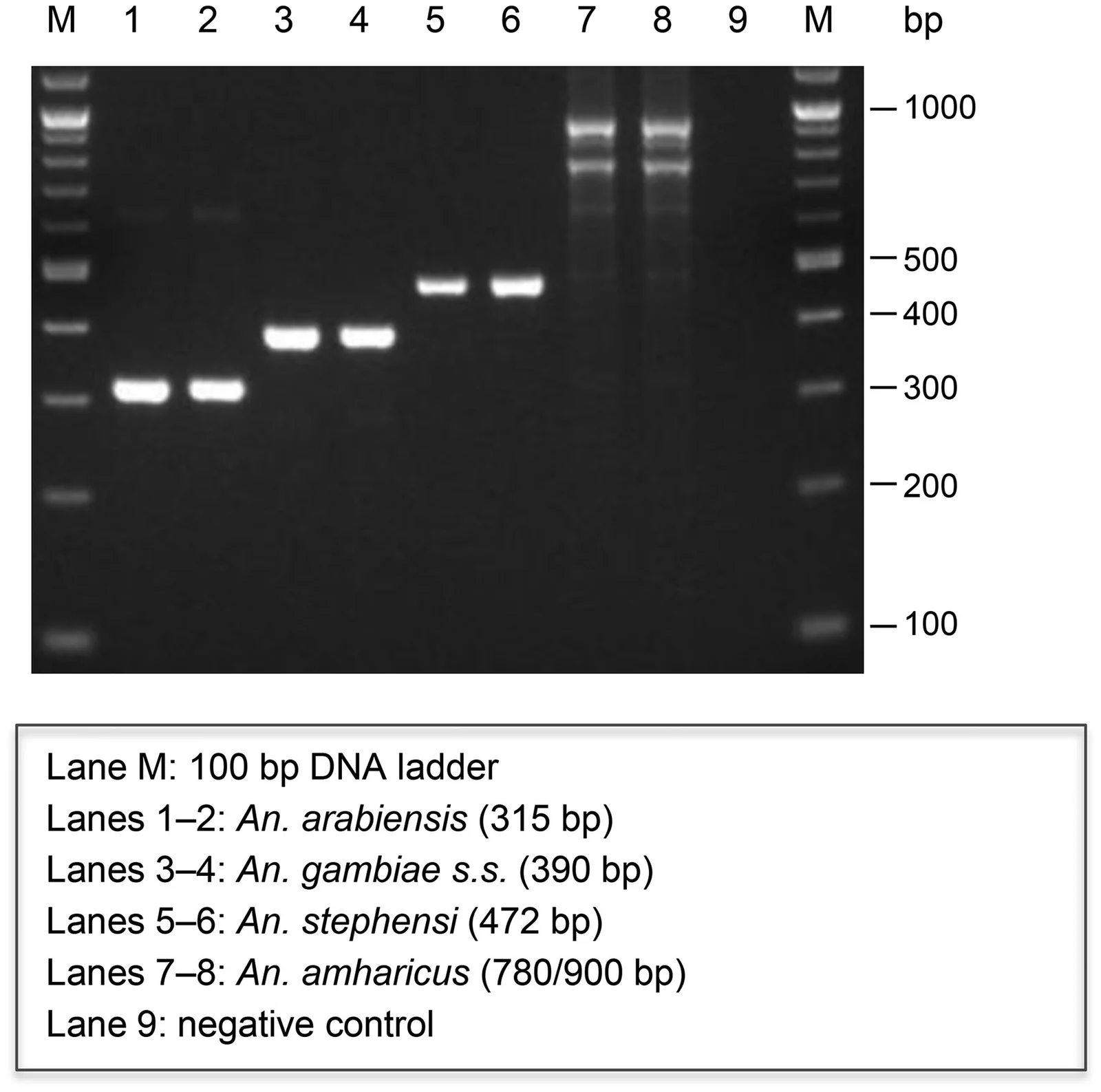
Multiplex PCR assay for species identification of *Anopheles stephensi* and members of the *Anopheles gambiae* complex in southern Ethiopia.

*Anopheles gambiae* s.s. was detected at 4 of the 8 prospective survey sites: Soddo, Birbir, Dila, and Arba Minch (Table 1; Fig. 1). All 40 *An. gambiae* s.s. specimens were concordantly identified by multiplex PCR and qPCR. *Anopheles arabiensis* was detected at all 8 sites, whereas *An. stephensi* was detected at 4 sites: Arba Minch, Dila, Hawassa, and Shashemene. *Anopheles gambiae* s.s. and *An. stephensi* occurred within the same geographic survey sites at Dila and Arba Minch.

### Molecular Identification of Archived Adult Mosquitoes

The 337 archived adult mosquitoes from Arjo and Gambella were analyzed separately from the prospective larval collections. Multiplex PCR identified 239 specimens as *An. arabiensis* and 93 as *An. amharicus*, while 5 remained unresolved. At Arjo, 93 of 145 archived specimens were identified as *An. amharicus* and 52 as *An. arabiensis*. At Gambella, 187 of 192 specimens were identified as *An. arabiensis*, with 5 unresolved. Selected archived *An. amharicus* and *An. arabiensis* specimens were subsequently included in sequence-based analyses.

### ITS2 Sequence and Phylogenetic Analyses

A total of 97 specimens representing *An. gambiae* s.s. (*n* = 40), *An. arabiensis* (*n* = 26), and *An. amharicus* (*n* = 31) yielded ITS2 sequences suitable for analysis (Supplementary Table S4). ITS2 sequences were identical within each of the 3 species groups, and representative sequences were deposited in GenBank as PZ496781 (*An. gambiae* s.s.), PZ496782 (*An. amharicus*), and PZ496783 (*An. arabiensis*).

All 40 *An. gambiae* s.s. specimens from Soddo, Dila, Birbir, and Arba Minch yielded identical ITS2 sequences, represented by accession PZ496781. The Ethiopian *An. gambiae* s.s. sequence was identical to reference sequences from Kenya and Tanzania and grouped with these sequences in the maximum-likelihood analysis with 81% bootstrap support (Fig. 3).

**Fig. 3.**
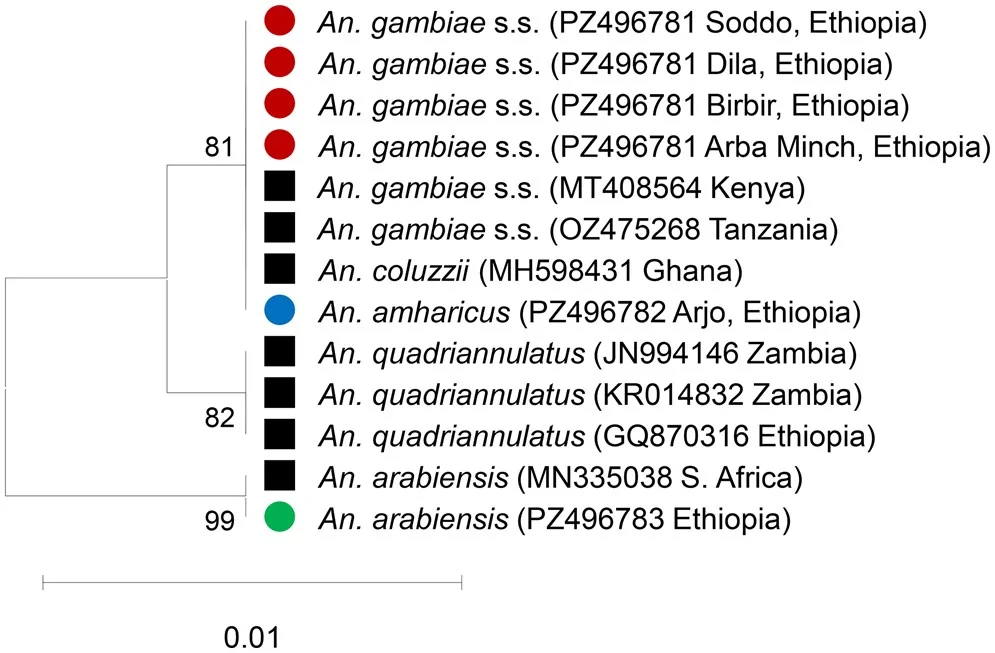
Maximum-likelihood phylogenetic tree inferred from representative ITS2 sequences of the *Anopheles gambiae* complex. The Jukes-Cantor nucleotide substitution model was selected based on the lowest BIC and AICc values. Branch support was assessed using 1,000 bootstrap replicates, and only bootstrap values ≥70% are shown. Ethiopian *An. gambiae* s.s. sequences generated in this study are indicated by red diamonds, *An. amharicus* by blue circles, and *An. arabiensis* by green symbols; black symbols indicate reference sequences retrieved from GenBank. Ethiopian *An. gambiae* s.s. grouped with reference sequences from Kenya and Tanzania. The scale bar represents 0.01 nucleotide substitutions per site.

The *An. amharicus* ITS2 sequence from Arjo (PZ496782) differed from the *An. gambiae* s.s. sequence by a single-base indel but showed no nucleotide substitutions across comparable sites (K2P distance = 0; Supplementary Table S5). The *An. gambiae* s.s. sequence differed from the *An. arabiensis* sequence by a K2P distance of 0.0115, whereas its distance from the *An. quadriannulatus* reference sequence was 0.0038 (Supplementary Table S5). Thus, ITS2 supported the identification of the Ethiopian *An. gambiae* s.s. specimens through sequence identity with East African *An. gambiae* s.s. reference sequences, but provided limited resolution among some closely related members of the *An. gambiae* complex.

### *cox1* Sequence Diversity and Haplotype Relationships

The same 97 specimens were subjected to mitochondrial *cox1* sequencing. Following sequence quality control, 84 sequences were retained, comprising 27 *An. gambiae* s.s., 26 *An. arabiensis*, and 31 *An. amharicus* specimens (Supplementary Table S4). Thirteen *An. gambiae* s.s. sequences were excluded because of insufficient sequence quality. The retained sequences comprised 10 unique haplotypes for *An. gambiae* s.s., 10 unique haplotypes for *An. arabiensis*, and 10 unique haplotypes for *An. amharicus*.

The TCS haplotype network revealed mitochondrial differentiation among the 3 molecularly assigned taxa (Fig. 4). *Anopheles amharicus* haplotypes formed a group separated by multiple mutational steps from most *An. gambiae* s.s. and *An. arabiensis* haplotypes. In contrast, *An. gambiae* s.s. and *An. arabiensis* showed closer mitochondrial relationships, with comparatively limited separation between their haplotypes. Pairwise K2P distances similarly showed greater mitochondrial differentiation involving the sampled *An. amharicus* haplotypes than was evident from ITS2 (Supplementary Table S5).

**Fig. 4.**
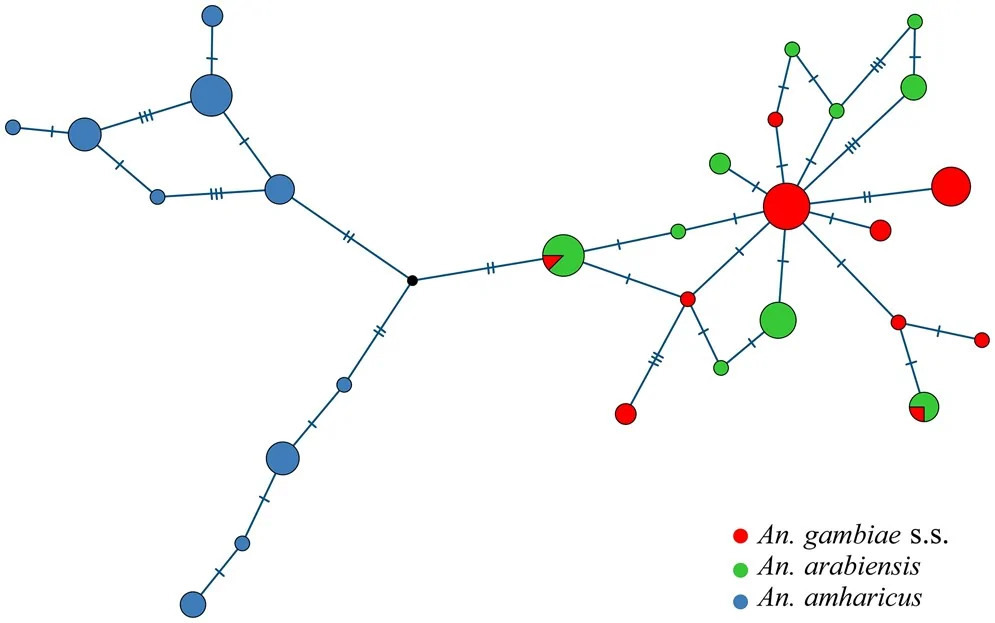
TCS haplotype network based on mitochondrial *cox1* sequences of *An. gambiae* s.s., *An. arabiensis*, and *An. amharicus*. Each circle represents a unique haplotype, with circle size proportional to haplotype frequency. Colors indicate species, and hatch marks on connecting branches represent mutational steps. *Anopheles amharicus* haplotypes showed greater mitochondrial differentiation from most *An. gambiae* s.s. and *An. arabiensis* haplotypes, whereas *An. gambiae* s.s. and *An. arabiensis* showed closer mitochondrial relationships.

### *cox1* Maximum-Likelihood Phylogeny

Maximum-likelihood analysis of the unique *cox1* haplotypes provided complementary information on mitochondrial relationships among the 3 taxa (Fig. 5). The sampled *An. amharicus* haplotypes from Arjo formed a mitochondrial grouping separated from most *An. gambiae* s.s. and *An. arabiensis* haplotypes, with ∼79% bootstrap support. In contrast, *An. gambiae* s.s. and *An. arabiensis* haplotypes were interspersed within the lower portion of the phylogeny, and most internal nodes separating these taxa received weak bootstrap support. Together, the *cox1* haplotype network and maximum-likelihood analysis differentiated the sampled Arjo *An. amharicus* haplotypes more clearly than ITS2, but cox1 did not consistently resolve *An. gambiae* s.s. from *An. arabiensis*.

**Fig. 5.**
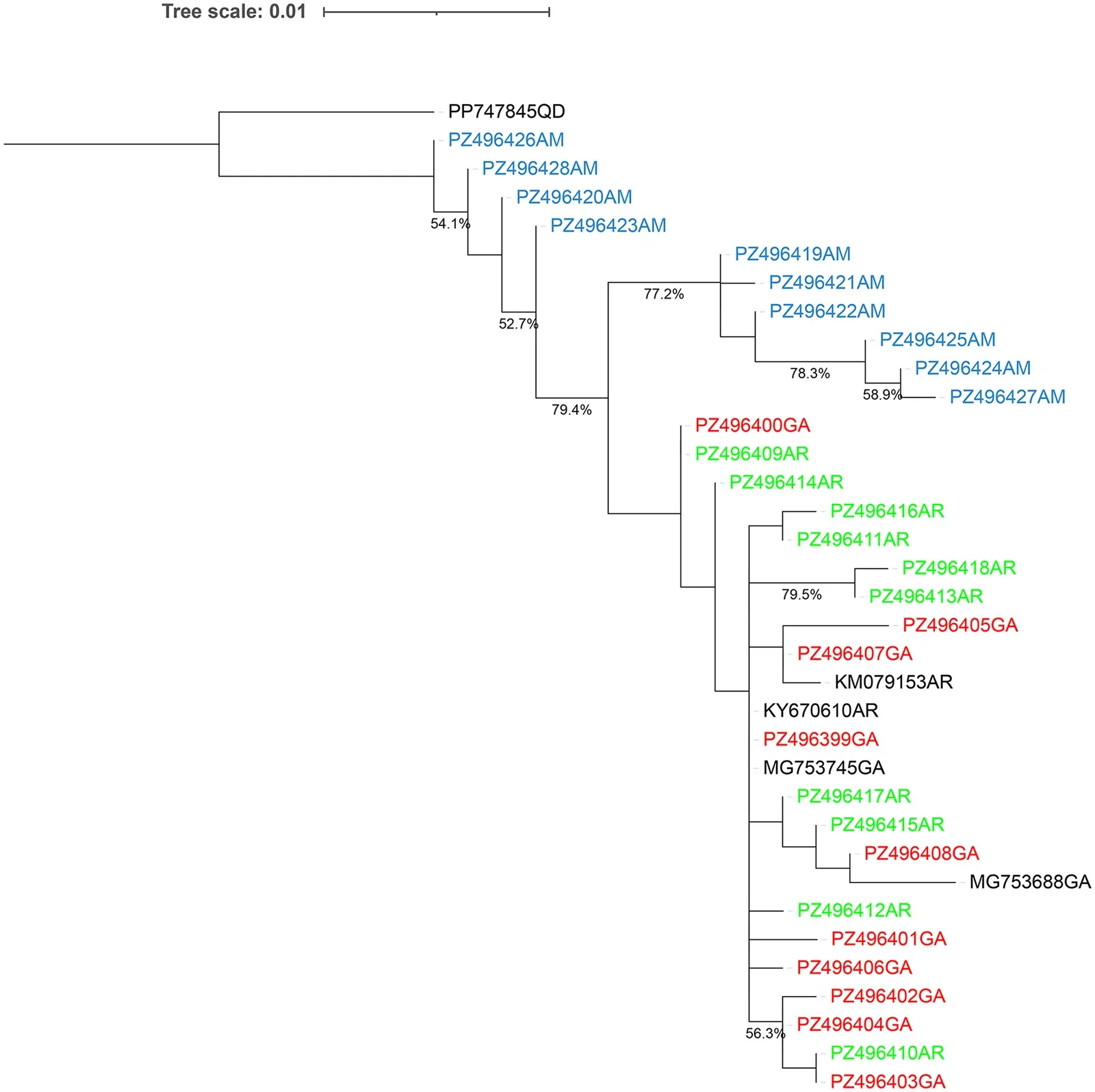
Maximum-likelihood phylogenetic tree inferred from mitochondrial *cox1* haplotypes of *Anopheles gambiae s.s., An. arabiensis*, and *An. amharicus*. Ethiopian haplotypes generated in this study are shown in red (GA; *An. gambiae* s.s.), green (AR; *An. arabiensis*), and blue (AM; *An. amharicus*); black labels indicate reference sequences retrieved from GenBank. Phylogenetic reconstruction was performed using PhyML under the HKY+I nucleotide substitution model, which was selected by ModelTest-NG according to both the BIC and AICc. Nucleotide frequencies and the proportion of invariant sites were estimated from the data. Branch labels indicate bootstrap support based on 1,000 replicates. *Anopheles quadriannulatus* (QD; PP747845) was used as the outgroup. The scale bar represents 0.01 nucleotide substitutions per site.

### Habitat-Level Distribution of Molecularly Identified Species

Of the 94 aquatic habitats represented in the molecular dataset, 40 were classified as rural, 17 as peri-urban, and 37 as urban (Table 2). *Anopheles gambiae* s.s. was detected in 18 habitats, including 12 rural and 6 peri-urban habitats, and was not detected in the molecularly sampled urban habitats. *Anopheles stephensi* was detected in 35 habitats, including 2 rural, 6 peri-urban, and 27 urban habitats. *Anopheles arabiensis* was detected across all 3 environmental settings (Table 2).

**Table 2.** Habitat-level detection of molecularly identified *Anopheles* species by environmental setting

Environmental setting	Rural	Peri-urban	Urban
**Molecularly sampled habitats**	40	17	37
***An. gambiae* s.s. positive, *n***	12	6	0
**Habitats positive, %**	30	35.3	0
***An. arabiensis* positive, *n***	25	5	15
**Habitats positive, %**	62.5	29.4	40.5
***An. stephensi* positive, *n***	2	6	27
**Habitats positive, %**	5	35.3	73
**Unresolved present, *n***	14	4	2
**Present, %**	35	23.5	5.4
***An. gambiae* s.s. + *An. stephensi* codetected, *n***	0	0	0

*Anopheles gambiae* s.s. larvae were detected in several natural and human-modified aquatic habitat types, including puddles, drainage channels, irrigation-associated pools, concrete water reservoirs, and open ponds (Supplementary Fig. S1). Although *An. gambiae* s.s. and *An. stephensi* occurred at the same survey sites at Dila and Arba Minch, the 2 species were not codetected within any of the 94 molecularly sampled aquatic habitats (Table 2).

Because larvae were subsampled to maximize habitat representation and sampling effort was not standardized among habitats, these habitat-level observations were treated descriptively rather than as estimates of species abundance or habitat preference.

## Discussion

This study provides molecular confirmation of *An. gambiae* s.s. at 4 geographical locations in southern Ethiopia: Soddo, Birbir, Dila, and Arba Minch. Species identity was supported by concordant multiplex PCR and species-specific qPCR results for all 40 *An. gambiae* s.s. specimens and by ITS2 sequence identity with *An. gambiae* s.s. reference sequences from East Africa. These findings extend previous reports of *An. gambiae* s.s. in Ethiopia and, together with recent genomic evidence from western Ethiopia, add to evidence that multiple members of the *An. gambiae* complex occur across different regions of the country (Kinfe and Tekie 2019, Gemechu et al. 2026). The present study does not establish the regional prevalence or relative abundance of *An. gambiae* s.s.; rather, its principal contribution is confirmation of the species at multiple southern Ethiopian locations using complementary molecular approaches.

Historically, *An. arabiensis* has been regarded as the predominant member of the *An. gambiae* complex and a major malaria vector in Ethiopia (Adugna et al. 2021). Nevertheless, previous molecular evidence documented *An. gambiae* s.s. in south-central Ethiopia (Kinfe and Tekie 2019), and recent whole-genome sequencing confirmed populations in Gambella and Benishangul-Gumuz in western Ethiopia (Gemechu et al. 2026). Thus, the present findings should not be interpreted as the first detection of *An. gambiae* s.s. in Ethiopia. Rather, confirmation at 4 geographically separated southern locations adds evidence that the species occurs in multiple regions of the country. Whether these southern populations are long established but incompletely documented, or reflect more recent changes in geographic distribution, cannot be determined from the present study.

The concordance among molecular approaches strengthens the taxonomic assignment of the southern Ethiopian specimens. All 40 *An. gambiae* s.s. specimens were concordantly identified by multiplex PCR and qPCR, and their ITS2 sequences were identical across the 4 collection sites and matched *An. gambiae* s.s. reference sequences from Kenya and Tanzania. The ITS2 phylogeny grouped these Ethiopian specimens with the East African *An. gambiae* s.s. references with 81% bootstrap support. However, the sequence comparisons also demonstrate limitations of ITS2 for resolving some closely related members of the *An. gambiae* complex. The representative *An. amharicus* ITS2 sequence from Arjo differed from the *An. gambiae* s.s. sequence by a single-base indel but showed no nucleotide substitutions across comparable sites, resulting in a K2P distance of zero. Moreover, this indel was not unique to *An. amharicus*. Consequently, ITS2 alone does not provide sufficient resolution to distinguish all closely related taxa represented in this study, emphasizing the importance of interpreting sequence information together with species-specific molecular assays.

Mitochondrial *cox1* analysis provided complementary information on genetic relationships among the molecularly assigned taxa. Both the TCS haplotype network and maximum-likelihood phylogeny showed greater differentiation of the sampled *An. amharicus* haplotypes from most *An. gambiae* s.s. and *An. arabiensis* haplotypes. In contrast, *An. gambiae* s.s. and *An. arabiensis* haplotypes were not consistently separated in the *cox1* phylogeny, and several relationships between these taxa had weak bootstrap support. The K2P analyses similarly demonstrated mitochondrial variation within and among the sampled taxa. These findings show that cox1 provides useful complementary information but should not be considered an independently diagnostic marker for all members of the *An. gambiae* complex. More generally, the differing resolution provided by ITS2 and *cox1* illustrates the limitations of inferring species relationships or population history from 1 or 2 loci.

The occurrence of *An. gambiae* s.s. in southern Ethiopia is also biogeographically plausible given the species’ established distribution elsewhere in East Africa. The identical ITS2 sequence observed among the 40 specimens from 4 southern Ethiopian sites and its identity with reference sequences from Kenya and Tanzania demonstrate sequence similarity at this locus but provide little information about population origin or connectivity. Likewise, mitochondrial *cox1*, as a single maternally inherited locus, cannot resolve the demographic history of these populations. The recent availability of whole-genome data for *An. gambiae* s.s. from western Ethiopia provides an important basis for future comparisons (Gemechu et al. 2026). Genome-wide sequencing of southern Ethiopian specimens, together with populations from western Ethiopia and neighboring East African countries, will be necessary to investigate population structure, gene flow, demographic history, and whether southern and western Ethiopian populations share a common evolutionary origin.

Habitat-level observations in the present study identified differences in the environments in which the molecularly identified species were detected, although these patterns should be interpreted as descriptive and exploratory. *Anopheles gambiae* s.s. was detected in 18 of the 94 molecularly sampled aquatic habitats, including rural and peri-urban habitats but not the sampled urban habitats. In contrast, *An. stephensi* was detected mainly in urban and peri-urban habitats. Previous studies in Ethiopia have similarly documented frequent occurrence of *An. stephensi* in artificial water-storage containers and other human-associated habitats in urban environments, although the species can also use natural habitats and occur in peri-urban and rural settings (Balkew et al. 2021, Emiru et al. 2023, Massebo et al. 2024, Zhou et al. 2024, Degefa et al. 2025). At Dila and Arba Minch, *An. gambiae* s.s. and *An. stephensi* occurred within the same geographic survey sites but were not codetected within the same molecularly sampled aquatic habitats. This observation is compatible with differences in larval habitat use but does not, by itself, demonstrate habitat preference or ecological partitioning.

Interpretation of these habitat patterns is limited by the surveillance sampling design. Larvae were subsampled from positive habitats to maximize habitat representation rather than to provide standardized estimates of abundance, and sampling effort was not equivalent among all habitat types and sites. Individual larvae collected from the same aquatic habitat also cannot be regarded as independent ecological observations, and some *Anopheles*-positive habitats were not represented in the molecular subsample. Consequently, differences in the numbers or proportions of molecularly identified larvae should not be interpreted as estimates of species prevalence, relative abundance, or habitat preference. The habitat-level comparisons reported here therefore provide hypotheses for subsequent investigation rather than formal evidence of ecological segregation. Standardized longitudinal surveys that use aquatic habitats as the observational units and account for site, season, habitat availability, and repeated sampling will be required to test habitat associations and patterns of coexistence among these vector species.

The molecular findings also highlight the value of incorporating molecular species identification into entomological surveillance in Ethiopia. Morphological identification cannot reliably distinguish sibling species within the *An. gambiae* complex, whereas molecular assays can provide the taxonomic resolution needed to characterize changes in vector composition. Molecular confirmation has similarly been important in documenting the distribution of invasive *An. stephensi* in Ethiopia (Balkew et al. 2020, Carter et al. 2021, Emiru et al. 2023). Integrating targeted molecular identification with existing larval and adult surveillance could therefore improve species-distribution mapping and facilitate recognition of changes in vector communities. Molecular diagnostics should complement, rather than replace, appropriately designed and standardized field surveillance.

The confirmation of *An. gambiae* s.s. at multiple sites in southern Ethiopia may be relevant to malaria vector surveillance because this species is an important malaria vector elsewhere in sub-Saharan Africa and may differ from *An. arabiensis* in behavior, ecology, and responses to vector-control interventions. However, the present study examined primarily larval occurrence and species identity and did not evaluate adult density, biting behavior, host preference, *Plasmodium* infection, vector competence, or insecticide susceptibility of the southern Ethiopian *An. gambiae* s.s. populations. Their contribution to local malaria transmission therefore remains unknown. This distinction is important because detection of a competent vector species does not establish its epidemiological importance at a particular location. Future studies combining molecular identification with longitudinal adult surveillance, parasite detection, host-feeding and biting-behavior analyses, and insecticide-resistance phenotyping will be needed to determine the role of these populations in malaria transmission and their relevance to existing vector-control interventions.

In conclusion, complementary molecular analyses confirmed *An. gambiae* s.s. at 4 geographically separated locations in southern Ethiopia and add to accumulating evidence of geographically broader species diversity within the *An. gambiae* complex in Ethiopia. ITS2 and *cox1* analyses supported the molecular characterization of the specimens while also demonstrating limitations in the resolution of closely related taxa. Habitat-level observations identified differences in the environments in which *An. gambiae* s.s. and *An. stephensi* were detected, but these patterns require confirmation using standardized ecological sampling. Expanded molecular surveillance, combined with genome-wide population analyses and studies of adult behavior, infection status, and insecticide susceptibility, will be important for defining the distribution, population structure, and epidemiological significance of *An. gambiae* s.s. in Ethiopia.

## Supplementary Material

tjag173_Supplementary_Data

## Data Availability

The ITS2 and *cox1* sequences generated in this study have been deposited in GenBank under accession numbers PZ496781–PZ496783 for ITS2 and accession numbers PZ496399–PZ496428 for *cox1*.
