# Supplementary material for: Molecular confirmation of Anopheles gambiae sensu stricto (Diptera: Culicidae) at multiple locations in southern Ethiopia
Source: J Med Entomol. 2026 Nov 12;63(6):tjag173. doi: 10.1093/jme/tjag173 (PMC13759216; doi:10.1093/jme/tjag173)
Supplement: Supplementary file 1 [file tjag173_supplementary_data.pdf]

Supplemental Materials

Supplemental Table S1. Primer and probe sequences used in this study.

No.	Primer/probe name	Sequence (5′→3′)	Reference
**1**	UN	GTGTGCCCCTTCCTCGATGT	Scott et al. (1993)
**2**	AR	AAGTGTCCTTCTCCATCCTA	Scott et al. (1993)
**3**	GA	CTGGTTTGGTCGGCACGTTT	Scott et al. (1993)
**4**	QDB	AGTGTCCAATGTCTGTGAAG	Haileselassie et al. (2021)
**5**	ITS2A	TGTGAACTGCAGGACACAT	Beebe and Saul (1995)
**6**	28SC	GTCTCGCGACTGCAACTG	Ali et al. (2022)
**7**	ITS2B	TATGCTTAAATTCAGGGGGT	Beebe and Saul (1995)
**8**	LCO1490	GGTCAACAAATCATAAAGATATTGG	Folmer et al. (1994)
**9**	HCO2198	TGATTTTTTGGTCACCCTGAAGTTTA	Folmer et al. (1994)
**10**	623F	GTGAAGCTTGGTGCGTGCT	Walker et al. (2007)
**11**	788R	GCACGCCGACAAGCTCA	Walker et al. (2007)
**12**	stq_F	TCTTTCCTCGCATCCAGTTG	Singh et al. (2023)
**13**	stq_R	CGGGAGAAGCGGTGATAAAT	Singh et al. (2023)
**14**	AR_FAM	TAGGATGGAGAAGGACACTTA-FAM	Walker et al. (2007)
**15**	AG_NED	CGGTATGGAGCGGGACACGTA-NED	Walker et al. (2007)
**16**	ST_VIC	CGTGCTAACCTCACTCACCCACAC-VIC	Singh et al. (2023)

Supplemental Table S2. Sampling characteristics and effort for prospective larval surveys at eight sites.

Site	First survey date	Last survey date	Minimum elevation (m)	Maximum elevation (m)	Environmental settings represented	Aquatic habitats surveyed	*Anopheles*-positive habitats	Habitats represented in molecular analysis	Larvae molecularly tested
Soddo	2025-11-30	2026-05-01	1822.1	2073.8	Peri-urban, Rural, Urban	48	24	15	58
Birbir	2026-05-02	2026-05-02	1159.8	1245.0	Rural	16	7	6	41
Arba Minch	2026-05-03	2026-05-04	1168.9	1380.5	Peri-urban, Rural, Urban	35	26	16	70
Dila	2025-12-01	2025-12-01	1413.6	1529.2	Peri-urban, Rural, Urban	24	8	8	35
Hawassa	2025-11-27	2026-04-30	1669.7	1740.8	Peri-urban, Rural, Urban	70	27	27	170
Shashemene	2025-11-29	2025-11-29	1894.8	2036.9	Rural, Urban	20	6	6	26
Bule Hora	2025-12-02	2025-12-02	1797.2	1813.2	Peri-urban	11	4	4	20
Jimma	2026-05-10	2026-05-12	1690.8	1787.7	Rural	12	12	12	83

Supplemental Table S3. Concordance between multiplex PCR and TaqMan qPCR among 503 prospectively collected larvae.

Multiplex PCR result	qPCR result	Final molecular assignment	n
An. gambiae	*An. gambiae*	*An. gambiae*	40
An. arabiensis	*An. arabiensis*	*An. arabiensis*	214
An. stephensi	*An. stephensi*	*An. stephensi*	155
Inconclusive	*An. arabiensis*	*An. arabiensis*	2
Inconclusive	*An. stephensi*	*An. stephensi*	3
Inconclusive	Inconclusive	Unresolved	89

Supplemental Table S4. Specimen information and GenBank accession numbers for ITS2 and *cox1* sequences generated in this study.

Specimen ID	Site	Species	ITS2 accession	*cox1* accession	*cox1* QC status
SD009.1	Soddo	*An. gambiae*	PZ496781	PZ496401	Retained
SD009.2	Soddo	*An. arabiensis*	PZ496783	PZ496411	Retained
SD009.3	Soddo	*An. gambiae*	PZ496781	PZ496399	Retained
SD315.1	Soddo	*An. gambiae*	PZ496781	PZ496399	Retained
SD315.2	Soddo	*An. gambiae*	PZ496781	PZ496401	Retained
SD315.3	Soddo	*An. gambiae*	PZ496781	PZ496399	Retained
SD315.4	Soddo	*An. gambiae*	PZ496781	PZ496400	Retained
SD315.5	Soddo	*An. gambiae*	PZ496781	PZ496401	Retained
SD317.1	Soddo	*An. gambiae*	PZ496781	PZ496401	Retained
SD317.2	Soddo	*An. gambiae*	PZ496781	PZ496402	Retained
SD317.3	Soddo	*An. gambiae*	PZ496781	PZ496403	Retained
SD317.4	Soddo	*An. gambiae*	PZ496781	PZ496399	Retained
SD317.5	Soddo	*An. gambiae*	PZ496781		Excluded after *cox1* sequence QC
SD318.1	Soddo	*An. gambiae*	PZ496781	PZ496399	Retained
SD318.2	Soddo	*An. gambiae*	PZ496781	PZ496401	Retained
SD318.3	Soddo	*An. gambiae*	PZ496781	PZ496401	Retained
SD318.4	Soddo	*An. gambiae*	PZ496781	PZ496399	Retained
SD318.5	Soddo	*An. gambiae*	PZ496781	PZ496404	Retained
SD321.1	Soddo	*An. gambiae*	PZ496781	PZ496405	Retained
SD321.2	Soddo	*An. gambiae*	PZ496781	PZ496406	Retained
SD321.3	Soddo	*An. gambiae*	PZ496781	PZ496405	Retained
SD321.4	Soddo	*An. gambiae*	PZ496781	PZ496407	Retained
SD321.5	Soddo	*An. gambiae*	PZ496781	PZ496399	Retained
SD323.1	Soddo	*An. gambiae*	PZ496781	PZ496401	Retained
SD331.1	Soddo	*An. gambiae*	PZ496781	PZ496399	Retained
SD331.2	Soddo	*An. gambiae*	PZ496781	PZ496408	Retained
SD331.3	Soddo	*An. arabiensis*	PZ496783	PZ496412	Retained
SD331.4	Soddo	*An. arabiensis*	PZ496783	PZ496409	Retained
SD331.5	Soddo	*An. arabiensis*	PZ496783	PZ496412	Retained
SD336.1	Soddo	*An. gambiae*	PZ496781	PZ496399	Retained
SD336.5	Soddo	*An. gambiae*	PZ496781	PZ496406	Retained
SD539.1	Soddo	*An. gambiae*	PZ496781		Excluded after *cox1* sequence QC
SD539.4	Soddo	*An. gambiae*	PZ496781		Excluded after *cox1* sequence QC
SD539.5	Soddo	*An. gambiae*	PZ496781		Excluded after *cox1* sequence QC
SD539.6	Soddo	*An. gambiae*	PZ496781		Excluded after *cox1* sequence QC

AM551.1	Arba Minch	*An. gambiae*	PZ496781		Excluded after *cox1* sequence QC
AM553.3	Arba Minch	*An. gambiae*	PZ496781		Excluded after *cox1* sequence QC
AM561.4	Arba Minch	*An. gambiae*	PZ496781		Excluded after *cox1* sequence QC
BB541.2	Birbir	*An. gambiae*	PZ496781		Excluded after *cox1* sequence QC
BB542.3	Birbir	*An. gambiae*	PZ496781		Excluded after *cox1* sequence QC
BB543.7	Birbir	*An. gambiae*	PZ496781		Excluded after *cox1* sequence QC
BB545.2	Birbir	*An. gambiae*	PZ496781		Excluded after *cox1* sequence QC
DL358.2	Dila	*An. arabiensis*	PZ496783	PZ496409	Retained
DL361.1	Dila	*An. gambiae*	PZ496781	PZ496399	Retained
DL340.6	Dila	*An. gambiae*	PZ496781		Excluded after *cox1* sequence QC
Arjo.001	Arjo	*An. arabiensis*	PZ496783	PZ496413	Retained
Arjo.002	Arjo	*An. arabiensis*	PZ496783	PZ496410	Retained
Arjo.003	Arjo	*An. arabiensis*	PZ496783	PZ496409	Retained
Arjo.004	Arjo	*An. amharicus*	PZ496782	PZ496419	Retained
Arjo.005	Arjo	*An. amharicus*	PZ496782	PZ496420	Retained
Arjo.006	Arjo	*An. arabiensis*	PZ496783	PZ496414	Retained
Arjo.007	Arjo	*An. arabiensis*	PZ496783	PZ496410	Retained
Arjo.008	Arjo	*An. arabiensis*	PZ496783	PZ496411	Retained
Arjo.012	Arjo	*An. amharicus*	PZ496782	PZ496420	Retained
Arjo.013	Arjo	*An. arabiensis*	PZ496783	PZ496409	Retained
Arjo.015	Arjo	*An. amharicus*	PZ496782	PZ496419	Retained
Arjo.016	Arjo	*An. amharicus*	PZ496782	PZ496419	Retained
Arjo.017	Arjo	*An. amharicus*	PZ496782	PZ496419	Retained
Arjo.018	Arjo	*An. amharicus*	PZ496782	PZ496421	Retained
Arjo.019	Arjo	*An. arabiensis*	PZ496783	PZ496413	Retained
Arjo.021	Arjo	*An. arabiensis*	PZ496783	PZ496415	Retained
Arjo.023	Arjo	*An. amharicus*	PZ496782	PZ496422	Retained
Arjo.035	Arjo	*An. arabiensis*	PZ496783	PZ496410	Retained
Arjo.036	Arjo	*An. amharicus*	PZ496782	PZ496420	Retained
Arjo.037	Arjo	*An. amharicus*	PZ496782	PZ496421	Retained
Arjo.038	Arjo	*An. amharicus*	PZ496782	PZ496422	Retained
Arjo.039	Arjo	*An. arabiensis*	PZ496783	PZ496409	Retained
Arjo.040	Arjo	*An. arabiensis*	PZ496783	PZ496416	Retained
Arjo.042	Arjo	*An. arabiensis*	PZ496783	PZ496413	Retained
Arjo.043	Arjo	*An. amharicus*	PZ496782	PZ496423	Retained
Arjo.045	Arjo	*An. amharicus*	PZ496782	PZ496419	Retained
Arjo.046	Arjo	*An. amharicus*	PZ496782	PZ496419	Retained

Arjo.047	Arjo	*An. amharicus*	PZ496782	PZ496419	Retained
Arjo.048	Arjo	*An. amharicus*	PZ496782	PZ496419	Retained
Arjo.049	Arjo	*An. amharicus*	PZ496782	PZ496420	Retained
Arjo.050	Arjo	*An. amharicus*	PZ496782	PZ496422	Retained
Arjo.057	Arjo	*An. amharicus*	PZ496782	PZ496422	Retained
Arjo.058	Arjo	*An. arabiensis*	PZ496783	PZ496411	Retained
Arjo.059	Arjo	*An. amharicus*	PZ496782	PZ496420	Retained
Arjo.060	Arjo	*An. amharicus*	PZ496782	PZ496424	Retained
Arjo.061	Arjo	*An. amharicus*	PZ496782	PZ496425	Retained
Arjo.062	Arjo	*An. amharicus*	PZ496782	PZ496426	Retained
Arjo.065	Arjo	*An. amharicus*	PZ496782	PZ496424	Retained
Arjo.066	Arjo	*An. amharicus*	PZ496782	PZ496427	Retained
Arjo.067	Arjo	*An. amharicus*	PZ496782	PZ496424	Retained
Arjo.069	Arjo	*An. arabiensis*	PZ496783	PZ496411	Retained
Arjo.071	Arjo	*An. amharicus*	PZ496782	PZ496424	Retained
Arjo.072	Arjo	*An. amharicus*	PZ496782	PZ496424	Retained
Arjo.074	Arjo	*An. arabiensis*	PZ496783	PZ496417	Retained
Arjo.076	Arjo	*An. arabiensis*	PZ496783	PZ496418	Retained
Arjo.080	Arjo	*An. arabiensis*	PZ496783	PZ496409	Retained
Arjo.083	Arjo	*An. amharicus*	PZ496782	PZ496426	Retained
Arjo.089	Arjo	*An. arabiensis*	PZ496783	PZ496411	Retained
Arjo.090	Arjo	*An. amharicus*	PZ496782	PZ496428	Retained
Arjo.091	Arjo	*An. amharicus*	PZ496782	PZ496426	Retained
Arjo.093	Arjo	*An. arabiensis*	PZ496783	PZ496411	Retained
Arjo.096	Arjo	*An. arabiensis*	PZ496783	PZ496409	Retained

A total of 97 specimens were sequenced for both markers: 40 *An. gambiae* s.s., 26 *An. arabiensis*, and 31 *An. amharicus*. All 97 ITS2 sequences were retained. For *cox1*, 84 sequences passed QC (27 *An. gambiae* s.s., 26 *An. arabiensis*, 31 *An. amharicus*); 13 *An. gambiae* s.s. sequences were excluded because sequence quality was insufficient.

Identical ITS2 sequences within each species were represented by a single GenBank accession: PZ496781 for *An. gambiae* s.s., PZ496782 for *An. amharicus*, and PZ496783 for *An. arabiensis*.

Supplemental Table S5. Summary of Kimura two-parameter (K2P) genetic distances for ITS2 and mitochondrial *cox1*.

Marker	Comparison	Basis	Mean K2P distance	Minimum	Maximum	Notes
ITS2	*An. gambiae s.s.* vs *An. amharicus*	Representative sequences	0.000000	0.000000	0.000000	1-base indel; gaps excluded from K2P
ITS2	*An. gambiae s.s.* vs *An. arabiensis*	Representative sequences	0.011484	0.011484	0.011484	Gap differences reported separately
ITS2	*An. gambiae s.s.* vs *An. quadriannulatus*	Representative sequences	0.003805	0.003805	0.003805	Gap differences reported separately
cox1	*Within An. gambiae s.s.*	45 pairwise comparisons	0.004469	0.001517	0.009175	Study haplotypes only
cox1	*Within An. arabiensis*	45 pairwise comparisons	0.005178	0.001517	0.009162	Study haplotypes only
cox1	*Within An. amharicus*	45 pairwise comparisons	0.008477	0.001517	0.015386	Study haplotypes only
cox1	*An. gambiae s.s.* vs *An. arabiensis*	100 pairwise comparisons	0.004875	0.000000	0.009175	Study haplotypes only
cox1	*An. gambiae s.s.* vs *An. amharicus*	100 pairwise comparisons	0.012902	0.006098	0.020095	Study haplotypes only
cox1	*An. arabiensis* vs *An. amharicus*	100 pairwise comparisons	0.013300	0.006098	0.020095	Study haplotypes only

K2P distances quantify nucleotide substitutions. Alignment gaps were excluded from distance calculations; therefore, indel differences in ITS2 are reported separately. *cox1* summaries are based on the 10 unique study haplotypes for each taxon included in the 35-sequence phylogenetic dataset.

Supplemental Figures

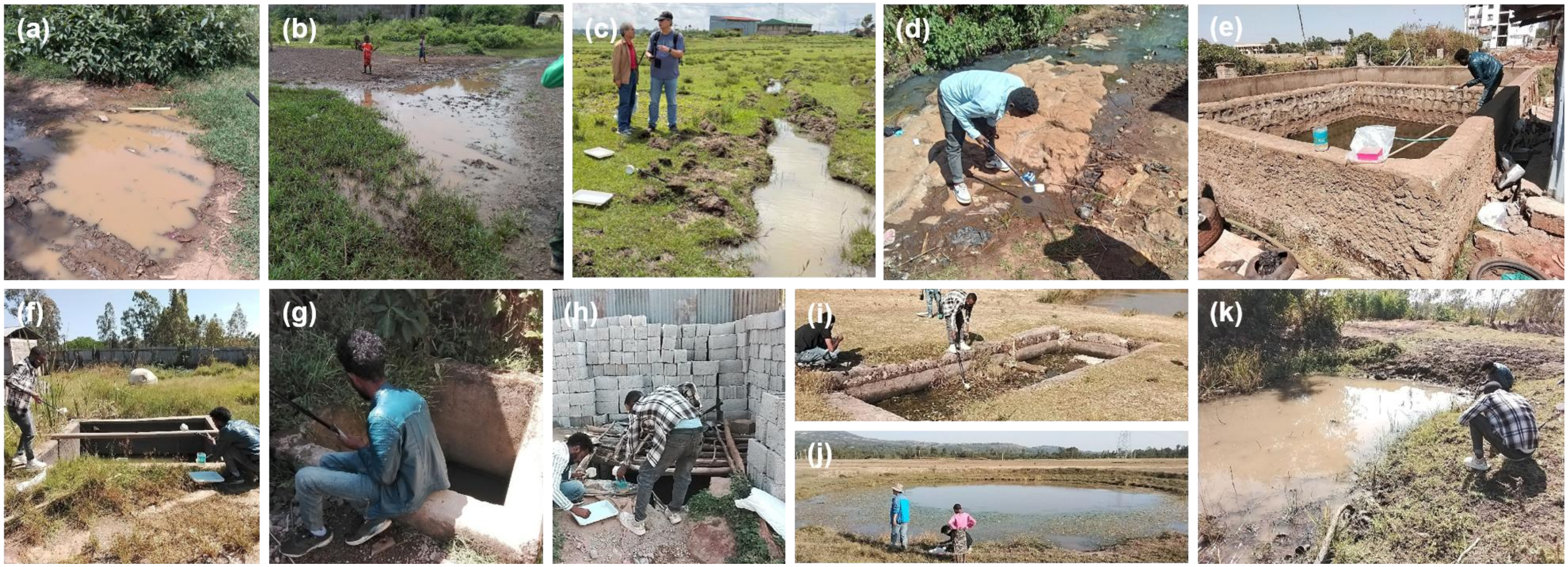

Supplemental Fig. S1. Representative natural and human-modified larval habitats where *An. gambiae* s.s. was detected, including puddles (a, b), drainage channels (c, d), concrete water reservoirs (e–h), irrigation-associated pools (i, j), and open ponds (k).
